# A modular tissue-clearing framework integrated with light-field microscopy enables rapid volumetric phenotyping of cardiac tissue

**DOI:** 10.21203/rs.3.rs-8566379/v1

**Published:** 2026-01-16

**Authors:** Shi Lin, Denise Schichines, Hua-Man Hsu, Yen-Ling Hung, Kaushik Chowdhury, I-Ting Lin, Chieh-Ju Wang, Min-Ju Tsai, Zi-yuan Liu, Kai-Chien Yang, Shih-Lei (Ben) Lai, Bi-Chang Chen, Rebecca Heald

**Affiliations:** 1 Department of Molecular and Cell Biology, University of California, Berkeley, Berkeley, CA, USA; 2 Department of Plant and Microbial Biology, University of California, Berkeley, CA, USA; 3 Biological Imaging Facility, University of California, Berkeley CA, USA; 4 Division of Research Microscopy Solutions, Carl Zeiss Co., Ltd., Hsinchu, Taiwan; 5 Department and Graduate Institute of Pharmacology, National Taiwan University College of Medicine, Taipei, Taiwan; 6 Institute of Biomedical Sciences, Academia Sinica, Taipei, Taiwan; 7 Graduate Institute of Life Sciences, National Defense Medical University, Taipei, Taiwan; 8 Research Center for Applied Sciences, Academia Sinica, Taipei, Taiwan; 9 Department of Internal Medicine, National Taiwan University Hospital, Taipei, Taiwan

## Abstract

Tissue clearing has transformed volumetric imaging by improving optical access to thick tissues, yet most existing protocols remain rigid and fail to accommodate the biochemical diversity of different samples. Here, we introduce a question-oriented modular framework that enables flexible assembly of clearing and imaging pipelines tailored to specific biological objectives. By systematically optimizing each module to preserve endogenous fluorescence, antigenicity, and tissue integrity while achieving high transparency and protein retention, we demonstrate its use across diverse cardiac applications—development, infarction and regeneration, as well as immune and vascular mapping—showing that different questions require distinct module assemblies and parameters. Coupled with light-field microscopy (LFM), the workflow efficiently captures submillimeter sections with 30–100-fold smaller datasets and rapid computational reconstruction, enabling high-throughput quantitative volumetric analysis. Together, this modular framework and imaging integration provide a rational and practical foundation for adaptable and interoperable 3D analyses of regenerative, pathological, and comparative systems across vertebrate models.

## Introduction

Tissue clearing has emerged as an indispensable technique for spatial biology, enabling three-dimensional visualization of complex architectures in intact tissues at unprecedented resolution and depth^1–9^. Recent innovations, including solvent-based (hydrophobic) systems (e.g., uDISCO^10^, PEGASOS^3^), hydrophilic reagents (e.g., CUBIC-HistoVision^11^, SUT^12^), and hydrogel-based chemistries (e.g., mPACT^13^, SHIELD^14^), have expanded the versatility of tissue clearing across organs and model organisms. Despite these rapid methodological advances, most clearing protocols remain rooted in a “one-protocol-fits-all” paradigm that rarely accommodates the biochemical diversity of biological tissues^15^. Variations in cellular composition, extracellular matrix density, and endogenous pigments yield distinct physicochemical constraints that affect experimental design. Consequently, a protocol optimized for one tissue type or molecular target often performs suboptimally when applied elsewhere, underscoring the need for a more adaptable and hypothesis-driven strategy.

In parallel, the practical utility of tissue clearing is fundamentally tied—and often constrained—by the optical and operational demands of Light-Sheet Fluorescence Microscopy (LSFM), which remains the dominant modality for volumetric imaging of cleared specimens^16, 17^. While LSFM provides high resolution, low photobleaching, and deep optical penetration, its intrinsic requirements—meticulous sample embedding, refractive-index matching, and multi-angle light-sheet alignment—make routine imaging labor-intensive and time-consuming^15, 18, 19^. Furthermore, LSFM acquisitions of even moderate-sized specimens routinely generate hundreds of gigabytes to terabytes of data, necessitating specialized workstations and computational expertise that many translational laboratories do not possess^16, 20^. As a result, large-cohort phenotyping and routine protocol iteration often become limited by the logistical burden of LSFM-based imaging. To broaden access to cleared-tissue workflows, we explored Light-Field Microscopy (LFM)—a technology originally developed for sub-millimeter-scale live imaging^21^—as an alternative for volumetric imaging of cleared specimens. LFM captures the full light field in a single exposure, enabling snapshot volumetric acquisition with minimal mounting requirements and a dramatically reduced data footprint. By pairing this accessible imaging regime with a modular clearing pipeline, we overcome practical barriers that have historically limited the scalability and translational reach of tissue-clearing workflows.

Building on this rationale, we establish a question-oriented modular framework for tissue clearing designed to synergize with this high-speed imaging capability. This approach emphasizes defining the biological objective—whether visualizing a specific structure, cell type, or protein—and then rationally selecting the most suitable combination of clearing modules. By dissecting the workflow into discrete, interoperable steps—fixation, permeabilization, decolorization, staining, and refractive-index matching—researchers can systematically evaluate chemical trade-offs and tailor each step to their experimental needs. In this work, we demonstrate that this modular strategy not only ensures robust clearing across diverse biological scenarios but also establishes a rapid, data-efficient workflow when coupled with light-field microscopy. We achieve instantaneous volumetric reconstruction at a fraction of the data size required by conventional methods, greatly expanding the accessibility of high-throughput 3D imaging.

## Results

### On the Modularity of Cardiac Tissue Clearing

**Fig. 1 |:**
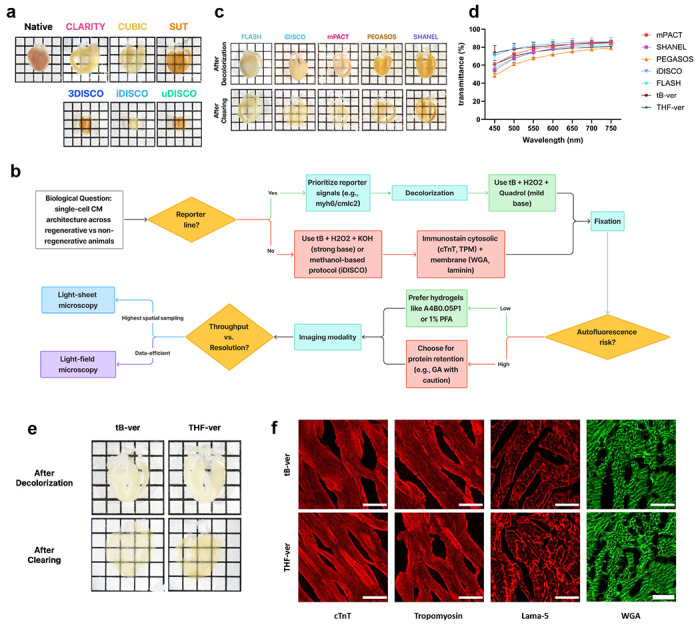
Establishing a question-oriented modular framework for cardiac tissue clearing. **a,** Bright-field images of adult C57BL/6 mouse heart slices (0.6 mm thick) treated with representative protocols (grid = 0.25 cm). **b,** Schematic workflow illustrating the modular design approach. **c,** Representative images of adult C57BL/6 mouse heart slices (0.6 mm thick) treated with the indicated decolorization reagents (top) and after CUBIC-1 clearing (bottom) (n = 6 per condition). **d,** Transmittance curves of cleared samples subjected to the indicated decolorization methods. **e,** Representative images of adult C57BL/6 mouse heart slices (0.6 mm thick) treated with tB- and THF-based decolorization reagents (top) and after CUBIC-1 clearing (bottom) (n = 6 per condition). **f,** Confocal images of IHC-stained heart samples treated with tB- (top) and THF-based (bottom) decolorization reagents, showing preserved antigen/protein integrity after decolorization (scale bars = 20 *μ*m).

To address the challenges of rendering dense, heme-rich cardiac tissue transparent, we first benchmarked six commonly used clearing protocols—PACT^8^, 3DISCO^2^, iDISCO^22^, uDISCO^10^, CUBIC^6^, and SUT^12^—on 0.6-mm-thick mouse heart slices, selected as a practical high-throughput careening format that preserves tissue-level architecture while accelerating chemical optimization. These methods revealed tissue-specific limitations: PACT induced notable distortion, DISCO variants caused substantial shrinkage, and while urea-based CUBIC and SUT achieved partial transparency, the slice interiors remained opaque even after prolonged incubation (Fig. 1a). Recent work have highlighted the value of modular frameworks in tissue clearing, allowing researchers to mix and match steps for customization based on sample type and goals^15, 23^. Building on this concept, we developed a question-oriented modular approach where the biological inquiry—such as visualizing cardiomyocyte architecture in disease models—guides the selection of optimized modules, including chemical reagents, genetic tools, or imaging strategies, to balance transparency with signal and structural preservation (Fig. 1b). For cardiac applications, we prioritized cytosolic (cardiac troponin T, tropomyosin) and membrane (Wheat Germ Agglutinin, laminin) markers commonly used in cardiovascular studies^24–28^.

**Extended Data Fig. 1 |:**
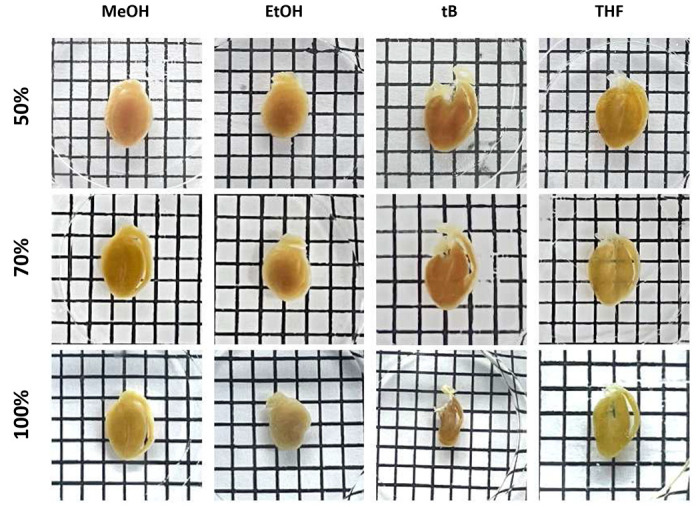
Solvent concentration–dependent effect on morphological changes and transparency. Representative images showing the effect of increasing solvent concentration (50%, 70%, and 100%) for methanol (MeOH), ethanol (EtOH), tert-butanol (tB), and tetrahydrofuran (THF). Hearts were immersed in each solvent for equal durations under identical conditions and visualized against a black-and-white grid background (grid spacing = 0.25 cm) to assess transparency and tissue shrinkage.

**Extended Data Fig. 2 |:**
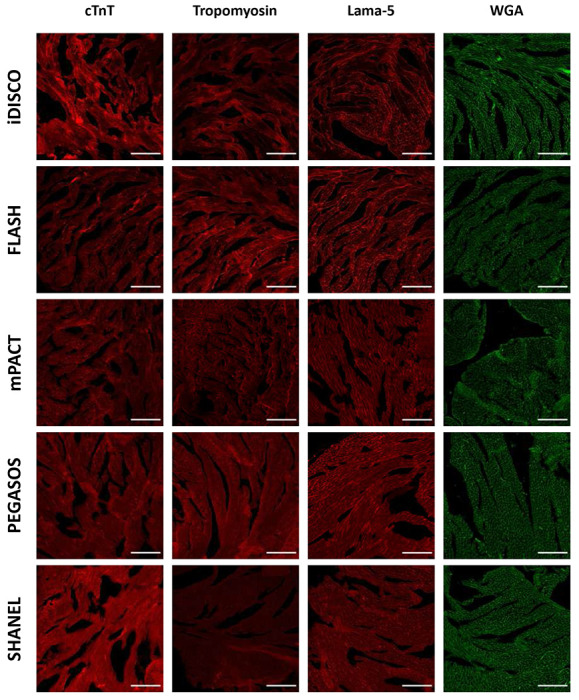
Antibody/dye compatibility test across different decolorization reagents. Representative confocal images of C57BL/6 mouse heart immunostained for cardiac troponin T (cTnT), tropomyosin, and laminin-5 (Lama-5), and lectin-labeled with wheat germ agglutinin (WGA) after decolorization with iDISCO, FLASH, mPACT, PEGASOS, or SHANEL. Scale bars, 200 *μ*m.

Given the high heme content in the cardiac tissue, we reasoned that effective decolorization was a critical initial step. We screened reagents from established protocols (FLASH^5^, iDISCO^22^, PEGASOS^3^, mPACT^13^, SHANEL^9^) to evaluate their efficacy in decolorizing cardiac tissue. Among these, the iDISCO reagents—containing hydrogen peroxide mixed with methanol—produced the most pronounced decolorizing effect and the highest transparency when followed by CUBIC-1 clearing (Fig. 1c,d). This suggested that hydrogen peroxide may be a key factor for effective decolorization in heme-rich cardiac tissue. Building on this observation and noting that cardiac samples became partially transparent in 50% alcohol (tert-butanol; tB) and ether (tetrahydrofuran; THF) solutions (Extended Data Fig. 1), we hypothesized that combining hydrogen peroxide with these solvents could further enhance decolorization. To test this, we formulated two peroxide–KOH cocktails (25% H_2_O_2_ and 0.05% KOH dissolved in 50% THF or tB in Milli-Q water). Transmittance analysis revealed that the tert-butanol–peroxide–KOH cocktail achieved transparency on par with iDISCO (Fig. 1d), and both our decolorizing cocktails and the iDISCO reagent effectively preserved the antigens and proteins of interest (Fig. 1e; Extended Fig. 2). However, our formulation avoids the tissue shrinkage commonly associated with the 100% methanol used in iDISCO, providing a more stable and tissue-friendly alternative for cardiac samples (Extended Data Fig. 1).

### Solvent gradient analysis reveals tert-butanol as the most fluorescence-compatible solvent for oxidative decolorization

**Fig. 2 |:**
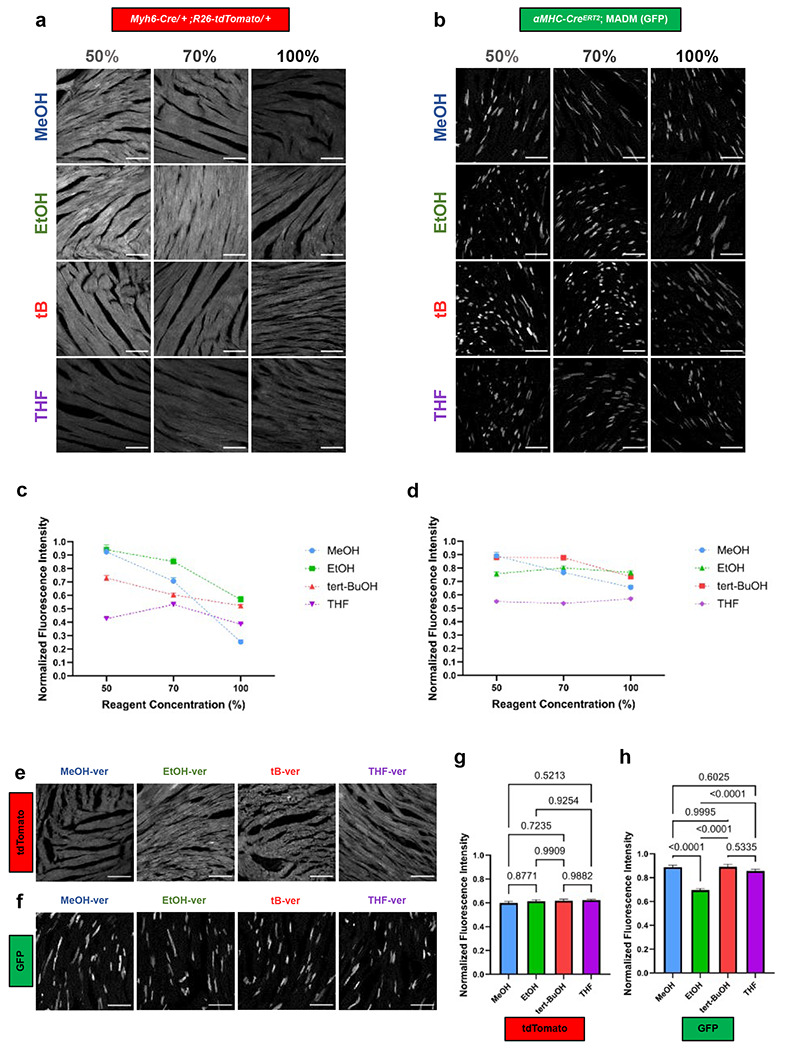
Stepwise quantitative screening identifies fluorescence-compatible solvent conditions for decolorization. **a,** Representative composite 3 × 4 montage of *Myh6-Cre/*+;*R26-tdTomato/*+ hearts showing TdTomato fluorescence after solvent treatment. Images were acquired using identical optical and detector settings. Columns indicate solvent type (methanol, ethanol, tert-butanol, tetrahydrofuran); rows indicate solvent concentration (50%, 70%, 100%). Scale bars, 200 *μ*m. **b,** Representative composite 3 × 4 montage of *αMHC-Cre*^*ERT*2^; MADM hearts showing GFP fluorescence after solvent treatment. Images were acquired using identical optical and detector settings. Columns indicate solvent type (methanol, ethanol, tert-butanol, tetrahydrofuran); rows indicate solvent concentration (50%, 70%, 100%). Scale bars, 200 *μ*m. **c,** Summary of TdTomato fluorescence intensity changes from panel (a). Line graph shows normalized fluorescence (F/F_0_) with error bars for each solvent and indicated concentration. Data represent n = 30 measurements per condition, collected from 3–4 hearts. **d,** Summary of GFP fluorescence intensity changes from panel (b). Line graph shows normalized fluorescence (F/F_0_) with error bars for each solvent and indicated concentration. Data represent n = 30 measurements per condition, collected from 3–4 hearts. **e,** Representative images showing TdTomato fluorescence preservation after solvent treatment with different solvent media. **f,** Representative images showing GFP fluorescence preservation after solvent treatment with different solvent media. **g-h,** Summary of normalized fluorescence intensity (F/F_0_) for TdTomato (left) and GFP (right) after decolorization in four solvent media combinations. All values are mean±SEM (n = 30 measurements per condition, collected from 3–4 hearts); the statistical significance was assess by one-way analysis of variance (ANOVA). Scale bars, 200 *μ*m.

To further guide solvent optimization in our modular framework and assess compatibility with endogenous fluorescence retention, we first noted that prior DISCO variants^2, 10, 22, 29^ and the related derivatives, including PEGASOS^3^, BALANCE^30^, and HYBRiD^23^, have predominantly relied on endpoint assessments after full gradient exposure to evaluate fluorescence preservation, potentially overlooking concentration-specific dynamics during progressive solvent exposure. To challenge this paradigm and capture gradient-dependent quenching profiles, we performed stepwise, isolated treatments at 50%, 70%, and 100% concentrations for methanol (MeOH), ethanol (EtOH), tert-butanol (tB), and tetrahydrofuran (THF), quantifying normalized fluorescence intensity (F/F_0_) relative to pre-exposure baselines in *Myh6-Cre/* + *;R26-tdTomato/* + and *αMHC-Cre^ERT2^*; MADM (GFP) cryosections (Fig. 2a,b).

Fluorescence quenching exhibited solvent-dependent nonlinearity. MeOH began with high retention at 50% (~0.92 F/F_0_) but dropped steeply to ~0.71 at 70% and ~0.25 at 100%, reflecting its strong dehydrating effect at higher concentrations. EtOH performed the best overall, maintaining consistently high fluorescence across the gradient (~0.94, ~0.85, and ~0.57 at 50%, 70%, and 100%, respectively). In contrast, t-BuOH exhibited moderate but notably stable retention (~0.73, ~0.60, and ~0.52), demonstrating reduced sensitivity to concentration changes compared with MeOH and EtOH. THF yielded the lowest fluorescence at 50% (~0.43) but showed a modest increase at 70% (~0.53) before declining again at 100% (~0.38). Collectively, these data highlight t-BuOH’s linear, concentration-tolerant behavior, in contrast to the sharp nonlinear declines observed in alcohols with higher dehydration rates (Fig. 2a,c).

GFP displayed parallel but generally attenuated trends. At 50%, MeOH and tB yielded the highest retention (~0.89 and ~0.87 F/F_0_, respectively), followed by EtOH (~0.75) and THF (~0.55), likely reflecting the stabilized hydrogen-bond networks in hydrated alcohols. At 70%, tB remained the strongest (~0.87), with EtOH close behind (~0.80), while MeOH dipped (~0.76) and THF plateaued (~0.53). At 100%, EtOH and tB again performed the best (~0.76 and ~0.73 F/F_0_), followed by MeOH (~0.65) and THF (~0.57) (Fig. 2b,d). Collectively, these profiles reveal early advantages for hydrophilic alcohols (MeOH, EtOH) under partially hydrated conditions but highlight tB as the most stable and predictable solvent across concentration gradients, with late-stage resilience for tB and THF in more anhydrous environments.

To further optimize decolorization for samples with endogenous fluorescence, we next tested peroxide–Quadrol cocktails containing 50% MeOH, EtOH, tB, or THF supplemented with 3% H_2_O_2_ and 3% Quadrol, which provides mild alkalinity (pH > 9) without the harsh denaturing effects of strong bases such as KOH. Under these conditions, tdTomato exhibited uniformly high stability, with all four solvents yielding similar fluorescence retention (MeOH ~0.59, EtOH ~0.61, t-BuOH ~0.62, THF ~0.62) (Fig. 2e,g), likely reflecting the intrinsic robustness of the tdTomato chromophore. In contrast, GFP showed modest solvent-dependent differences, with t-BuOH and MeOH performing slightly better and THF yielding comparable retention (Fig. 2f,g). These results indicate that, while peroxide–Quadrol treatment minimizes solvent-specific effects for tdTomato, t-BuOH still offers the most consistent performance across fluorophores when considering both endogenous fluorescence and the concentration-gradient experiments above. Together, these findings support t-BuOH as the preferred solvent backbone for oxidative decolorization in fluorescent cardiac samples.

### Hydrogel-based post-fixation preserves protein integrity and minimizes autofluorescence after decolorization

**Fig. 3 |:**
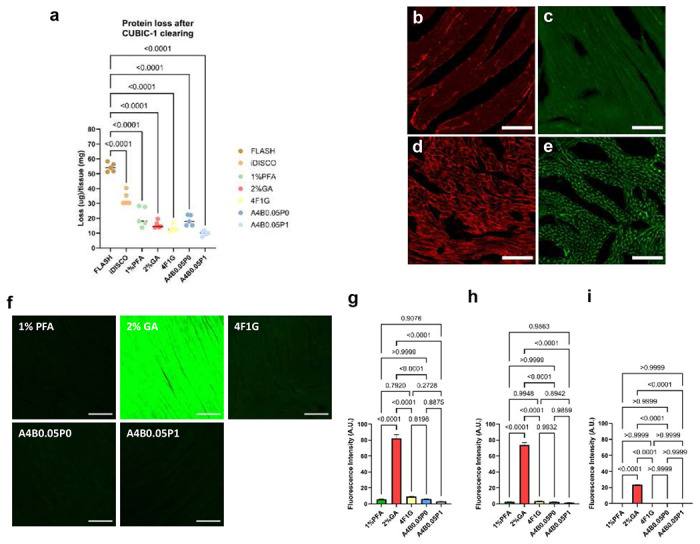
Establishing a question-oriented modular framework for cardiac tissue clearing. **a,** Quantification of protein loss after CUBIC clearing. Statistical significance was determined by two-tailed t-tests. n= 5 samples for each condition. **b,** Representative confocal image of laminin-stained heart samples without post-fixation. **c,** Representative confocal image of WGA-stained samples without post-fixation. **d,** Representative confocal image of laminin-stained samples with A4B0.05P1 post-fixation. **e,** Representative confocal image of WGA-stained samples after A4B0.05P1 post-fixation. **f,** Representative fluorescence images of heart samples post-fixed using indicated fixatives, acquired at an excitation wavelength of 488 nm. **g,** Quantification of autofluorescence levels measured at 488 nm. **h,** Quantification of autofluorescence levels measured at 568 nm. **i,** Quantification of autofluorescence levels measured at 647 nm. All values are mean±SEM (n = 30 measurements per condition, collected from 5 hearts); the statistical significance was assess by ANOVA. Scale bars, 200 *μ*m.

To mitigate protein loss during subsequent clearing steps, we evaluated post-fixation strategies on decolorized samples. Without any post-fixation, CUBIC-1 caused substantial protein leakage (mean loss ≈40–60 μg per tissue slices, n = 5; see “iDISCO/FLASH” range in Fig. 3a) as well as marked damage to membrane proteins/antigens (Fig. 3b,c). We therefore screened five post-fixation reagents—1% paraformaldehyde (PFA), 2% glutaraldehyde (GA), 4F1G (4% paraformaldehyde with 1% glutaraldehyde in PBS), A4B0.05P0 (4% acrylamide, 0.05% bisacrylamide and 0.25% VA-044 initiator in PBS), and A4B0.05P1 (4% acrylamide, 0.05% bisacrylamide, 1% PFA and 0.25% VA-044 initiator in PBS)—assessing not only their ability to preserve overall protein integrity but also potential side effects such as autofluorescence, which could interfere with downstream fluorescence imaging. Our evaluation prioritized compatibility with routine imaging channels (488 nm and 568 nm for visible light, 647 nm for near-infrared) to ensure minimal interference in multi-channel applications.

**Extended Data Fig. 3 |:**
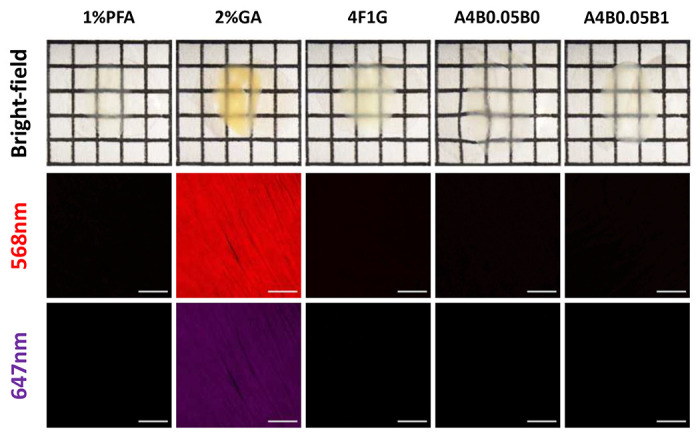
Effect of post-fixation reagents on tissue reddening and autofluorescence. Representative images of decolorized C57BL/6 mouse heart slices treated with different post-fixation reagents following CUBIC clearing. **Top:** Bright-field images showing tissue transparency and degree of reddening after post-fixation with 1% paraformaldehyde (PFA), 2% glutaraldehyde (GA), 4F1G, A4B0.05B0, or A4B0.05B1 (grid spacing = 0.25 cm). **Middle:** Representative fluorescence images of post-fixed samples acquired at an excitation wavelength of 568 nm to assess autofluorescence in the red channel. **Bottom:** Representative fluorescence images acquired at 647 nm to evaluate far-red autofluorescence. Only 2% GA induced elevated autofluorescence in the far-red channel. Scale bars, 20 *μ*m.

We found that post-fixatives exhibited wavelength-dependent autofluorescence: 2% GA produced strong signals across the measured spectral range, whereas 4F1G showed moderate fluorescence only at 488 and 568 nm. In contrast, 1% PFA and hydrogel-based fixatives (A4B0.05P0 and A4B0.05P1) exhibited negligible autofluorescence at 488 nm and none in the red or near-infrared channels (Fig. 3f–i). Both GA-containing conditions caused tissue reddening or yellowing that persisted after CUBIC clearing, underscoring the need for post-fixation following decolorization (Extended Fig. 3). Importantly, although low, even these “low-AF” fixatives exhibited nonzero background at 488 nm (typically ~3–10 A.U. for PFA/hydrogel conditions), indicating that they are not entirely background-free in the blue-green channel. Beyond fluorescence background, we next examined protein retention under each condition. Our protein-loss analysis revealed that A4B0.05P1, compared with 1% PFA and A4B0.05P0 (both of which also produced low autofluorescence), achieved higher preservation of overall protein content (Fig. 3a). Although 4F1G showed comparable performance, it tends to cause tissue yellowing (Extended Fig. 3). We therefore selected A4B0.05P1 as the post-fixative because it not only minimized autofluorescence and protein loss but also preserved membrane-associated proteins and antigens (Fig. 3d,e). This highlights the importance of incorporating a post-fixation step between decolorization and clearing to maintain their integrity.

### Light-field microscopy enables rapid, data-efficient volumetric imaging of cleared cardiac tissue

Having established a clearing pipeline that renders sub-millimeter cardiac sections transparent while preserving fluorescence and protein content, we next asked how best to image these samples in a way that balances spatial resolution with throughput and data volume. Light-sheet fluorescence microscopy (LSFM) has become a benchmark technique for tissue clearing, yet its implementation remains labor-intensive and computationally demanding^20^. Preparing samples requires agarose embedding, careful alignment within a refractive-index–matched chamber, and often multiple acquisition angles. Moreover, LSFM routinely generates hundreds of gigabytes per sample—even for sub-millimeter sections—and reconstruction typically requires hours on high-end or custom-built workstations^31^. These barriers limit throughput and accessibility, particularly for translational studies where large sample sets are needed.

**Fig. 4 |:**
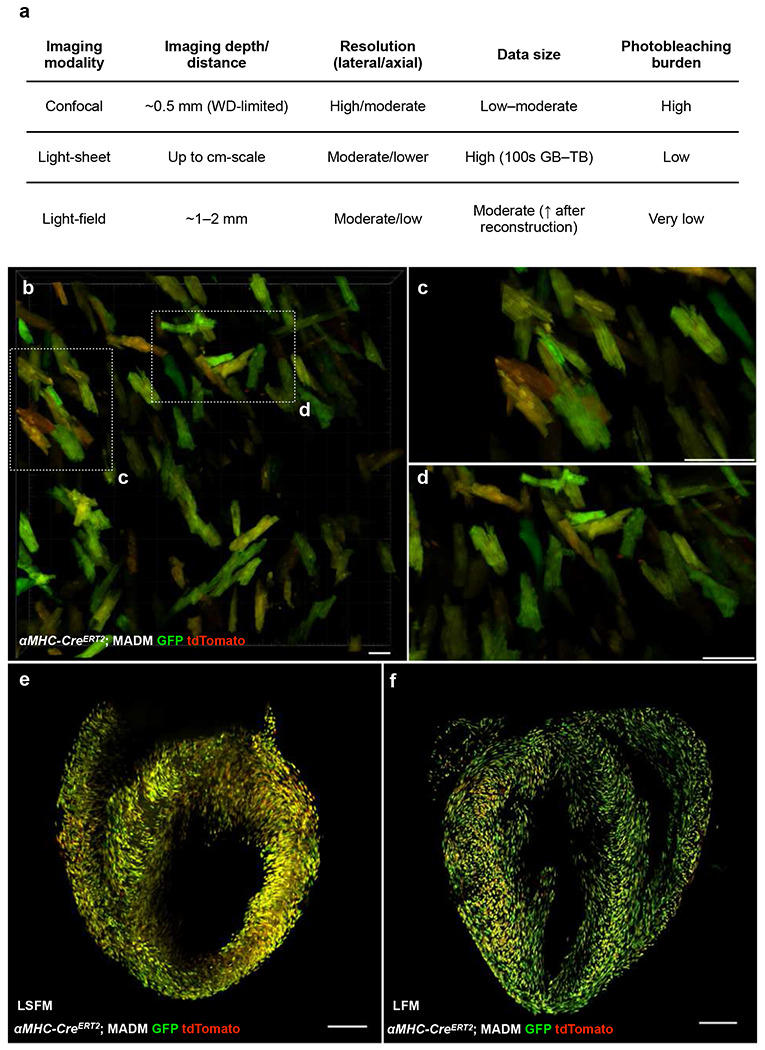
Light-field imaging of cleared cardiac tissue with imaging modality comparison. **a,** Comparison between confocal, light-sheet, and light-field microscopy across four practical parameters relevant to cleared-tissue imaging. **b,** Confocal image of an *αMHC-Cre*^*ERT*2^; MADM mouse heart showing high-contrast mosaic patterns of clonally related cardiomyocytes. Scale bar, 50 *μ*m. **c-d,** Magnified views of boxed regions in **b,** illustrating the presence of unicolor and dual-color cardiomyocytes characteristic of the MADM system. Scale bars, 20 *μ*m. **e,** Representative light-sheet fluorescence (LSFM) image of an *αMHC-Cre*^*ERT*2^; MADM mouse heart. Scale bar, 500 *μ*m. **f,** Representative light-field (LFM) image of an identically processed sample. Scale bar, 1 mm.

To address these constraints, we explored Light-Field Microscopy (LFM) as a high-throughput alternative for volumetric imaging of cleared tissue. Unlike scanning-based techniques, LFM captures both the spatial and angular distribution of emitted light through a microlens array, encoding a four-dimensional light field in a single exposure. Because the effective imaging depth of LFM in biological specimens has been reported to range from ~180 μm to ~600 μm in live^21, 32^, scattering tissues, we reasoned that cleared specimens—whose optical heterogeneity is substantially reduced—should permit reliable volumetric reconstruction across a modestly larger axial span. We therefore defined a practical “working thickness” of 0.5–1.0 mm for cleared cardiac sections, balancing the optical performance envelope of LFM with the need to resolve single-cell-level anatomy. Constraining the samples to this thickness eliminates the need for mechanical z-scanning, enabling instantaneous volumetric capture with dataset sizes typically <3 GB—comparable to confocal stacks—while preserving the spatial structural detail required for cardiac analysis (Fig. 4a).

**Extended Data Fig. 4 |:**
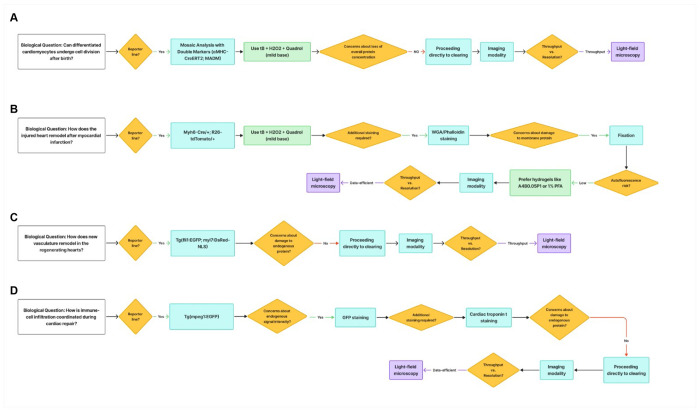
Context-specific assemblies for different biological questions. Decision flow diagrams illustrate how the modular clearing framework is adapted to four representative use-cases: **a,** Postnatal cardiomyocyte division (MADM). **b,** Structural remodeling after myocardial infarction (MI). **c,** Neo-vascularization during zebrafish heart regeneration. **d,** Macrophage infiltration during zebrafish cardiac repair.

As a proof-of-concept, we benchmarked LSFM and LFM using Mosaic Analysis with Double Markers (*αMHC-Cre^ERT2^*; MADM) mice^33^, which generate sparse, clonal labeling of cardiomyocytes through Cre-dependent interchromosomal recombination (Extended Data Fig. 4a). Following tamoxifen induction, dividing cardiomyocytes produce daughter cells labeled in red (tdTomato), green (GFP), both (yellow), or remain unlabeled, generating endogenous, spectrally distinct fluorescence at true single-cell resolution. This sparse clonal labeling not only provides well-separated cardiomyocytes whose geometries offer a stringent test for volumetric reconstruction, but also captures proliferative behavior relevant for cardiac-regeneration studies (Fig 4b–d). We performed side-by-side volumetric imaging using both modalities. LFM datasets (1.8–2.4 GB) were reconstructed within seconds on a standard workstation, whereas LSFM volumes exceeded 100 GB and required hours of acquisition and processing. Despite a ~50-fold reduction in data size, LFM preserved the structural fidelity needed to resolve cytosolic boundaries and distinguish clonal populations (Fig. 4e–f; Supplementary Movie 1). Snapshot acquisition further minimized photobleaching and enabled rapid multicolor interrogation across large sample sets. Thus, by aligning the ‘working thickness’ of our clearing protocol with the optical performance envelope of LFM, we establish a fast, data-efficient platform for high-throughput cardiac phenotyping. Together, these results demonstrate that light-field microscopy provides a fast, data-efficient, and accessible platform for volumetric imaging of cleared cardiac tissue, establishing the foundation for subsequent disease and comparative studies.

### Integrated clearing–light-field imaging enables rapid, multiscale visualization of cardiac injury and regeneration across vertebrates

Having established the feasibility of volumetric imaging in a reporter line model, we next applied the optimized clearing–imaging pipeline to disease-mimicking injury paradigms across species to demonstrate its biological and translational relevance. Heart failure remains the leading cause of mortality worldwide, driven largely by the heart’s limited regenerative capacity after ischemic injury^34, 35^. Following myocardial infarction (MI), the irreversible loss of cardiomyocytes leads to fibrotic scarring and ventricular remodeling, whereas certain lower vertebrates retain the ability to regenerate myocardium through coordinated cardiomyocyte proliferation, neovascularization, and immune modulation^36^. Developing an imaging framework that can capture these complex multicellular dynamics across species is therefore critical for understanding the mechanisms that underlie successful versus failed regeneration.

**Fig. 5 |:**
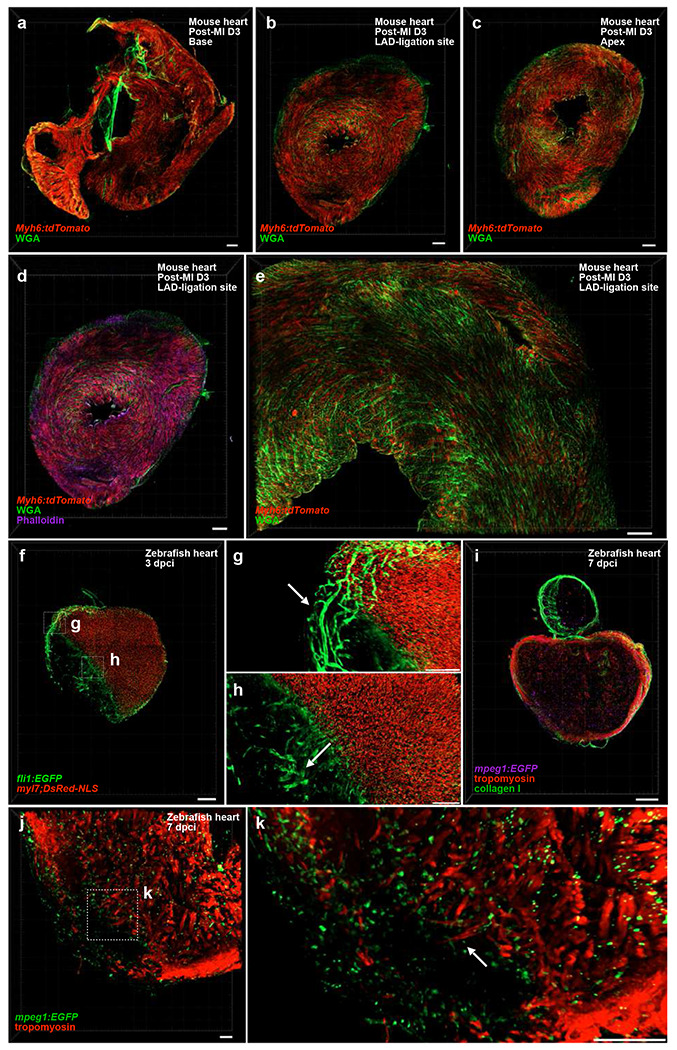
Cross-species visualization of injury models. **a–c,** Transverse sections of a myocardial infarction (MI)–treated postnatal day 21 (P21) *Myh6-Cre/+;R26-tdTomato/+* mouse heart from base to apex. Red, tdTomato (cardiomyocytes); green, wheat germ agglutinin (WGA; membranes). Scale bars, 500 *μ*m. **d,** Representative image of an MI-treated P21 *Myh6-Cre/+;R26-tdTomato/+* heart stained with phalloidin (purple) and WGA (green). Scale bar, 500 *μ*m. **e,** Close-up view of the infarct region showing cardiomyocyte disorganization and extracellular matrix remodeling. Red, tdTomato; green, WGA. Scale bar, 200 *μ*m. **f,** Representative sagittal section of a *Tg(fli1:EGFP; myl7:DsRed-NLS)* zebrafish heart. Red, myl7:DsRed-NLS (cardiomyocytes); green, fli1:EGFP (endothelium). Scale bar, 200 *μ*m. Boxed regions are magnified, with arrows showing the sprouting superficial **(g)** and intraventricular **(h)** coronary arteries. Scale bars, 100 *μ*m **i,** Representative sagittal section of a *Tg(mpeg1:EGFP)* zebrafish heart immunostained for tropomyosin (red) and collagen I (purple). Scale bar, 200 *μ*m. **j,** Higher-magnification view of the border zone showing cardiomyocytes (red) and infiltrating macrophages (green). The boxed region **(k)** highlights macrophage infiltration within the injury core, with arrow showing protruding cardiomyocyte. Scale bars, 50 *μ*m **(j)** and 100 *μ*m **(k)**.

We first applied this framework to the MI model in *Myh6-Cre/* + *;R26-tdTomato/* + mice (Extended Data Fig. 4b). In most preclinical studies, practical quantification relies on representative sections through the infarcted territory (e.g., apex to ligation site spanning ~4–6 mm for proximal LAD occlusions)^37^ using interval sampling strategies (e.g., 5 μm sections at 0.3-0.4 mm intervals)^38–41^, while prior whole-mount clearing approaches employed confocal microscopy on 600-μm-thick tissue slabs to image ROIs^42^. In contrast, multi-voxel LFM imaging delineated the infarct, border, and remote zones within a single dataset, revealing cytoskeletal disorganization and differential TdTomato signal intensities that mark viable versus necrotic cardiomyocytes (Fig. 5a–c). Representative samples were further stained with WGA and phalloidin to visualize extracellular matrix and actin filaments, respectively (Fig 5d). The ability to visualize these spatially distinct domains within minutes highlights the method’s efficiency in assessing structural remodeling after injury.

We next tested our pipeline on zebrafish cryoinjury models. In this system, the practical axial region of interest—particularly in widely used injury paradigms such as apex resection and cryoinjury—typically spans ~150–200 μm (reflecting the fact that these procedures remove or damage ~20% of the ventricle, whose total axial dimension is ~600–1000 μm) ^43, 44^. As a result, most regeneration studies either analyze representative thin cryosections (~10 μm) from this localized apical domain or rely on thicker cryosections (~60 μm)^45^ to preserve partial border-zone architecture for quantifying scar resolution, cardiomyocyte proliferation, and tissue regrowth. Although this targeted sampling balances efficiency with statistical robustness, it also introduces potential bias, as the precise sections selected and the region analyzed may vary across experiments^46^. Previous efforts to extend volumetric coverage using X-CLARITY and LSFM have likewise been limited by time-consuming workflows, incomplete depth penetration, and cumbersome reconstruction pipelines^47^. By adopting 200-μm vibratome sections—matching both the biologically relevant region of interest and the working-thickness regime of LFM—our pipeline enables rapid, full-volume visualization of regenerative dynamics in this critical tissue zone. Using this approach, we can resolve superficial and intraventricular coronary sprouts as well as proliferating cardiomyocytes, both of which are essential components of zebrafish heart regeneration using the *Tg(fli1:EGFP;myl7:DsRed-NLS)* fish (Fig. 5f–h; Extended Data Fig 4c; ; Supplementary Movie 2). Early and spatially coordinated revascularization is known to scaffold cardiomyocyte repopulation and is a prerequisite for successful repair^47, 48^; our workflow captures these events in their native 3D context with minimal imaging burden, providing a robust platform for iterative and reproducible phenotyping across large cohorts. Similarly, using *Tg(mpeg1:EGFP)* zebrafish, we mapped the spatial dynamics between macrophages and repopulating cardiomyocytes during regeneration. Our pipeline enabled rapid characterization of how immune-cell infiltration modulates the regenerating myocardium and coordinates with newly repopulated cardiomyocytes to remodel the extracellular matrix, highlighting another essential step in cardiac regeneration, where immune-driven modulation of the microenvironment supports cardiomyocyte repopulation^49^ (Fig. 5i–k; Extended Data Fig. 4d; Supplementary Movie 3).

**Fig. 6 |:**
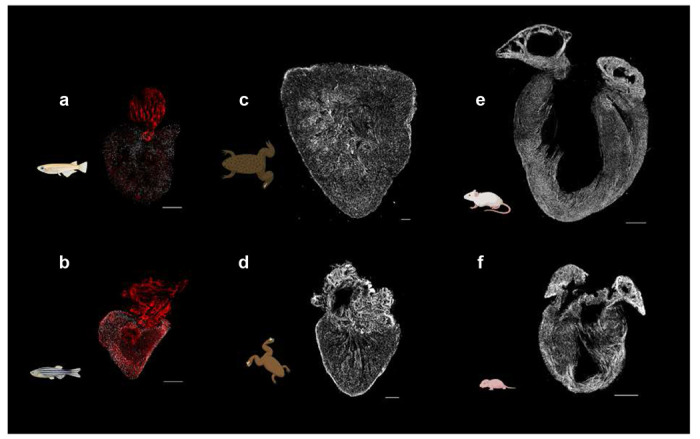
Phylogenetic comparison of myocardial architecture across major vertebrate clades. **a–b,** Representative hearts from *Tg(fli::GFP; zfmlc2 5.1k: DsRed2-nuc)* medaka (a) and *Tg(fli1:EGFP; myl7:DsRed-NLS)* zebrafish (b), highlighting endothelial networks in medaka and coronary arteries in zebrafish (red, *fli1:EGFP*) and cardiomyocyte nuclei (grey, *myl7:DsRed-NLS*). Scale bars, 200 *μ*m. **c-d,** Representative hearts from *Xenopus laevis* (c) and *Xenopus tropicalis* (d) stained with tropomyosin–AF568 (grey), revealing trabecular myocardial organization in in anuran amphibians. Scale bars, 500 *μ*m. **e-f,** Representative hearts from adult **(e)** and postnatal **(f)** *Myh6-Cre/+; R26-tdTomato/+* mice, labeled with tropomyosin–AF568 (grey), illustrating compact myocardial architecture in mammals. Scale bars, 1 mm

Finally, to assess the scalability of our modular pipeline for visualizing conserved and divergent features of myocardial architecture, we applied it phylogenetically across representative vertebrate models encompassing mammals, amphibians, and teleost fishes. Among teleosts, the *Tg(fli1:EGFP;myl7:DsRed-NLS)* zebrafish hearts displayed a conspicuous coronary artery network that was absent in the *Tg(fli::GFP; zfmlc2 5.1k: DsRed2-nuc)* medaka, underscoring the anatomical divergence between regenerative and non-regenerative fish (Fig. 6a–b). In amphibians, cTnT-stained *Xenopus tropicalis* and *X. laevis* showed densely trabeculated myocardium resembling that of teleost fishes (Fig. 6c–d). In mammals, both neonatal and adult *Myh6-Cre* hearts exhibited a compact and well-organized myocardial architecture (Fig. 6e–f).

## Discussion.

Our work establishes a question-oriented modular framework that redefines tissue clearing as a hypothesis-driven process rather than a fixed chemical recipe. Instead of selecting reagents by chemical type or legacy protocol, we begin with the biological question—such as resolving myocardial architecture, single-cell cardiomyocyte morphology and proliferation, or vascular remodeling—and assemble the clearing modules accordingly. This approach operationalizes the conceptual “modular pipeline” proposed in earlier reviews^15^, transforming it into a practical, adaptable strategy for diverse tissues and model organisms. The heart is an instructive test case: it combines dense, heme-rich myocardium, complex extracellular matrix, dynamic immune infiltration, and species-specific vascular and myocardial architectures. These intrinsic features are not merely descriptive—they determine the constraints that clearing pipelines must handle. High heme content and compact musculature, in particular, lead to substantial light absorption and scattering, and in mammalian models such as mouse, these properties make residual heme the dominant barrier to efficient clearing. Importantly, this constraint is not universal across vertebrates. In our dataset, only mouse hearts required an aggressive decolorization treatment, whereas Xenopus and teleost hearts—which contain less heme due to smaller size and have more trabecular myocardium—reached practical transparency without dedicated decolorization treatment. This species-dependent divergence highlights the need for a modular rather than fixed clearing strategy, in which tissue biochemistry dictates which modules are necessary and how they should be tuned (Supplementary Table). Within this modular logic, systematically evaluating the decolorization step for efficacy, antigen integrity, and fluorophore compatibility allowed us to identify tert-butanol–peroxide–base cocktails as a robust solution when decolorization is required. Other systems—such as neural tissue, where lipid extraction and myelination dominate the optical landscape—would invoke a different subset and ordering of modules, underscoring that our framework is tissue-guided rather than universally prescriptive.

Within this modular philosophy, distinct biological questions recruit different subsets and parameterizations of clearing modules. Analyses of extracellular matrix remodeling, immune-cell infiltration, cell-boundary integrity, muscle-fiber organization after injury, and coronary vasculature across animal models each impose unique constraints on epitope preservation, staining strategy, and workable imaging thickness. Our use cases illustrate this logic. In myocardial infarction scars imaged using the *Myh6-Cre/*+*;R26-tdTomato/*+ system, preserving membrane and extracellular-matrix markers is the primary priority and therefore requires carefully controlled post-fixation alongside efforts to minimize fixative-induced autofluorescence. In contrast, when working with *αMHC-Cre^ERT2^*; MADM hearts, maintaining endogenous fluorescence becomes the dominant constraint, and post-fixation is generally unnecessary unless additional membranous epitopes must be stabilized. Beyond these biochemical considerations, another practical consideration is the concept of a “working thickness” for light-field microscopy. Although tissue clearing reduces scattering and refractive-index heterogeneity, it does not alter the fundamental axial-resolution limits imposed by the objective lens and the encoded light-field optics. In our implementation, a 10× objective yields ~57-μm axial resolution with a ~1712-μm working distance, whereas a 25× water-immersion objective achieves ~2.8-μm axial resolution with a ~278-μm working distance, creating a trade-off between sample thickness and achievable axial resolution. As a result, the working thickness we propose is not a fixed number but depends on both specimen size and the spatial scale of the biological question. For example, although an entire zebrafish heart falls within the nominal working distance of the 10× configuration, an axial resolution of ~57 μm is insufficient to resolve immune-cell–cardiomyocyte interactions; yet the same configuration is adequate for quantifying cardiomyocyte regeneration in 0.6 mm MADM slabs, where relevant features are larger and more sparsely distributed. Together, these examples illustrate that context-specific biochemical and optical constraints jointly determine which modules—and parameterizations—are appropriate for a given application, reinforcing that our framework prioritizes question-oriented optimization rather than a single canonical ‘cardiac’ protocol.

The combination of a modular tissue-clearing framework with a commercially accessible LFM platform has practical implications for broadening access to volumetric cardiac imaging. By reducing data demands and simplifying sample mounting and acquisition, LFM lowers the infrastructural barriers that have historically restricted tissue clearing to specialized imaging labs. In translational and basic research settings, this approach enables rapid volumetric screening of injury models, regenerative phenotypes, and pharmacological responses. Importantly, our LFM-based workflow is not intended to replace LSFM or confocal microscopy, but to serve as a complementary front-end modality that rapidly surveys large tissue volumes, identifies regions of interest, and guides subsequent high-resolution LSFM or confocal acquisitions where finer axial detail is required. More broadly, we envision this work helping to ‘publicize’ tissue clearing by providing researchers with a rational framework for matching biochemical modules and accessible imaging regimes to diverse cardiac questions—and, by extension, to other organ systems where biochemical constraints and spatial readouts can be clearly defined.

## Methods.

### Animals.

#### Mouse care.

All mouse experiments were performed in accordance with institutional and national ethical guidelines and were approved by the Institutional Animal Care and Use Committees of the University of California, Berkeley, and the National Taiwan University College of Medicine (IACUC No. 20230191). Mice were housed under specific pathogen-free conditions with a 12h light/12 h dark cycle and provided ad libitum access to food and water. Both sexes were used for neonatal mice, and adult male hearts were included in the study. Experimental animals were assigned to groups using block randomization on a rolling basis to ensure adequate sample sizes for each condition. *Zebrafish care*. All zebrafish were bred and maintained in the fish facility at the Institute of Biomedical Sciences, Academia Sinica, under standard conditions (28.5 °C, 14 h light/10 h dark cycle). Animal care and experimental procedures were conducted in strict accordance with the Guide for the Care and Use of Laboratory Animals and with protocols approved by the Academia Sinica Institutional Animal Care and Use Committee (IACUC; Protocol ID: 18-12-1241). The protocol was reviewed and approved by the Committee on the Ethics of Animal Experiments of Academia Sinica. All surgeries were performed under tricaine anesthesia, and every effort was made to minimize animal suffering. *Frog care*. All *Xenopus laevis* and *X. tropicalis* frogs were used and maintained in accordance with standards established by the UC Berkeley Animal Care and Use Committee and approved in our Animal Use Protocol. Adult *Xenopus* used in this study were obtained from breeding colonies maintained at UC Berkeley. Frogs were kept in recirculating aquatic systems with monitored water quality and temperature, maintained at 20–23 °C for *X. laevis* and 23–26 °C for *X. tropicalis*, and fed Nasco frog brittle.

### PACT, CUBIC, SUT, 3DISCO, iDISCO, and uDISCO processing.

Hearts from 8–12-week-old wild-type C57BL/6 mice were anesthetized, excised, rinsed in phosphate-buffered saline (PBS), and fixed in 4% paraformaldehyde (PFA; Electron Microscopy Sciences, cat. no. 15710-S) at 4 °C for 48 h. Fixed samples were washed thoroughly in PBS and sectioned into 0.6 mm slices using a vibratome before clearing treatments. For **PACT**^8^, samples were embedded in A1P4 hydrogel solution (1% acrylamide [Bio-Rad, cat. no. 161-0140], 0.125% bisacrylamide [Bio-Rad, cat. no. 161-0142], 4% PFA, and 0.025% VA-044 initiator [Wako, cat. no. VA-044] (w/v) in 1× PBS) overnight at 4 °C, polymerized at 37 °C for 12 h, washed three times in PBS (1 h each), cleared in SDS/borate buffer (4% SDS in 0.2 M boric acid, pH 8.5) at 37 °C for 3 days, and immersed in 87% (vol/vol) glycerol in Milli-Q water at room temperature (RT) for 1 day. For **CUBIC**^6^, slices were incubated in ScaleCUBIC-1 buffer (25% [w/w] N,N,N′,N′-tetrakis(2-hydroxypropyl)ethylenediamine [Quadrol; Sigma-Aldrich, cat. no. 122262], 25% urea [Sigma-Aldrich, cat. no. U5378], and 15% Triton X-100 [Merck, cat. no. T9284] in Milli-Q water) for 3 days at 37 °C, then immersed in ScaleCUBIC-2 buffer (25% urea, 50% sucrose, 10% triethanolamine [Sigma-Aldrich, cat. no. 90279], balance H_2_O) for 1 day at RT. For **SUT**^12^, samples were incubated in SUT buffer containing 25% (wt/vol) urea, 15% (vol/vol) Triton X-100, and 8% (wt/vol) SDS at 37 °C for 3 days. For **3DISCO**^2^, slices were sequentially incubated in 50%, 70%, 80%, and 100% (vol/vol) tetrahydrofuran (THF; Sigma-Aldrich, cat. no. 186562-12X100ML) in Milli-Q water for 1 h each at RT, followed by 100% THF overnight, an additional 1 h in 100% THF, 1 h in dichloromethane (DCM; RT), and immersion in BABB (benzyl alcohol:benzyl benzoate = 1:2; Sigma-Aldrich, cat. nos. 24122 and W213802) for 1 week at RT. For **iDISCO**^22^, samples were dehydrated through 50%, 80%, and 100% methanol (in PBS) for 1 h each twice, bleached overnight at 4 °C in 5% H_2_O_2_ prepared in 20% DMSO/methanol (1 vol 30% H_2_O_2_ : 1 vol DMSO : 4 vol methanol, ice-cold), then rehydrated stepwise (100%, 80%, 50% methanol, PBS) and washed twice for 1 h in PBS/0.2% Triton X-100. Samples were delipidated in DCM (Sigma-Aldrich, cat. no. 270997-12X100ML) for 1 h at RT and immersed in BABB for 1 week at RT. For **uDISCO**^10^, slices were incubated sequentially in 50%, 70%, 80%, and 100% (vol/vol) tert-butanol (tB; Sigma-Aldrich, cat. no. 360538) in Milli-Q water for 1 h each at RT, then in 100% tB overnight, followed by an additional 1 h in 100% tB, 1 h in DCM at RT, and immersion in BABB for 1 week at RT.

### Decolorization.

0.6 mm vibratome-sectioned mouse heart slices were processed using the decolorization steps from representative clearing protocols under original conditions. For **FLASH**, slices were incubated overnight at room temperature (RT) in a decolorization solution containing dimethyl sulfoxide (DMSO), 30% hydrogen peroxide (H_2_O_2_), and PBS (1:2:3, v/v/v). For **mPACT**^13^, slices were incubated overnight at 37 °C in a solution of 1% α-thioglycerol (Sigma-Aldrich, cat. no. 88640), 4% sodium dodecyl sulfate (SDS), and 0.2 M boric acid in PBS. For **iDISCO**^22^, slices were sequentially dehydrated in 50% methanol in PBS (1 h, RT), 80% methanol (1 h, RT), and 100% methanol (1 h twice, RT), then bleached overnight at 4 °C in ice-cold 5% H_2_O_2_ prepared in 20% DMSO/methanol (1 vol 30% H_2_O_2_ : 1 vol DMSO : 4 vol methanol). Post-bleaching, slices were rehydrated stepwise in methanol (1 h twice, RT), 20% DMSO/methanol (1 h twice, RT), 80% methanol (1 h, RT), 50% methanol (1 h, RT), and PBS (1 h twice, RT). For **PEGASOS**^3^, slices were incubated overnight at 37 °C in a decolorization solution containing 25% (v/v) N,N,N′,N′-tetrakis(2-hydroxypropyl)ethylenediamine (Quadrol; Sigma-Aldrich, cat. no. 122262) and 5% (v/v) ammonium hydroxide in PBS. For **SHANEL**^9^, slices were sequentially treated at 37 °C as follows: 10% (w/v) CHAPS and 25% (w/v) N-methyldiethanolamine in PBS (1 h); 10% (w/v) Triton X-100 and 25% (w/v) N-methyldiethanolamine in PBS (1 h); and 200 mM SDS with 25% (w/v) N-methyldiethanolamine overnight on a shaking rocker. For **our optimized decolorization protocol**, slices were pre-incubated in 50% tert-butanol or tetrahydrofuran (THF) supplemented with 0.05% potassium hydroxide (KOH) in PBS for 30 min at RT, followed by decolorization in a solution containing 3% H_2_O_2_, 50% tert-butanol or THF, and 0.05% KOH in PBS for 30 min at RT. For samples retaining endogenous fluorescence, KOH was substituted with 3–5% Quadrol to minimize fluorophore quenching.

### Cryosectioning and immunohistochemistry.

Decolorized mouse hearts were washed thoroughly in PBS, cryoprotected in 30% (w/v) sucrose at 4 °C overnight, then equilibrated sequentially in 1:1 sucrose:OCT (Tissue-Tek, Sakura Finetek, Torrance, CA) for 6 h at 4 °C and 100% OCT overnight at 4 °C before being frozen and stored at −80 °C. Cryosections (10 μm thick) were collected for immunostaining. Slides were rehydrated in PBS, rinsed in distilled water, and subjected to heat-induced epitope retrieval in 10 mM citrate buffer (pH 6.0) for 15 min in a microwave at full power. Sections were washed thoroughly in 0.2% PBS–Tween 20, permeabilized in 0.5% PBS–Triton X-100, and blocked for 1 h at room temperature in 5% bovine serum albumin (BSA) and 5% normal goat serum in PBS. Primary antibodies included cardiac troponin T (CT3; DSHB, 1:100), tropomyosin (CH1; DSHB, 1:100), laminin-5 (L9393; Sigma-Aldrich, 1:2000), and wheat germ agglutinin (WGA; Thermo Fisher Scientific, 1:500). Secondary antibodies included goat anti-mouse IgG2a cross-adsorbed Alexa Fluor 568 (A-21134; Thermo Fisher Scientific, 1:400), goat anti-mouse IgG1 cross-adsorbed Alexa Fluor 568 (A-21124; Thermo Fisher Scientific, 1:400), and goat anti-rabbit IgG (H+L) cross-adsorbed Alexa Fluor 647 (A-21244; Thermo Fisher Scientific, 1:400). Primary antibodies were incubated overnight at 4 °C, followed by secondary antibody incubation for 3 h at room temperature.

### Evaluation of post-fixative–induced autofluorescence.

200 μm-thick PFA-fixed and decolorized heart slices were incubated in 1% PFA, 2% GA, or 4F1G at 4 °C overnight. Samples were washed thoroughly with PBS before confocal imaging. For hydrogel-embedded conditions (A4B0.05P0 and A4B0.05P1), samples were incubated in monomer solutions at 4 °C overnight, transferred to room temperature (RT) for 4 h, and then washed thoroughly in PBS prior to imaging. For each dataset, a maximum intensity projection over a 30 μm depth was generated from a representative field of view (approximately 850 μm × 850 μm) on the tissue surface.

### Evaluation of fluorescence quenching by solvent incubation and decolorization.

PFA-fixed *Myh6-Cre/* + *;R26-tdTomato/* + and *αMHC-Cre^ERT2^* ; MADM hearts were cryosectioned into 30 μm sections. Thirty sections per condition (total across three animals) were selected. TdTomato fluorescence was quantified in MYH6:TdTomato hearts, and GFP fluorescence was assessed in *αMHC-Cre^ERT2^*; MADM hearts. Sections were incubated with 50%, 70%, and 100% solutions of methanol, ethanol, tert-butanol, or tetrahydrofuran for solvent-induced quenching evaluation. For decolorization analysis, the same solvents were supplemented with 3% Quadrol to increase the pH above 9. In a final step, sections were treated with a decolorization reagent composed of 3% Quadrol and 3% H_2_O_2_ in 50% solvent (in Milli-Q water).

### Image processing and fluorescence quantification.

All fluorescence images were acquired using identical laser power, detector gain, and exposure settings across experimental groups. Z-stack datasets were processed in Zeiss ZEN black edition (Carl Zeiss) to generate maximum-intensity projections (MIPs). Quantitative analysis was conducted in Zeiss Arivis using either the Intensity Threshold Segmenter or Blob Finder modules, or both, to separate fluorescence signal from background noise. After segmentation, background pixels were excluded from further analysis, and the mean fluorescence intensity of all remaining signal-classified pixels within each image was computed. This value was used to represent the overall fluorescence level for that specimen. Mean signal-intensity profiles were automatically extracted from these processed images and exported for visualization and statistical analysis in GraphPad Prism (GraphPad Software).

### Protein Loss Measurement.

Vibratome sections (0.6 mm) were post-fixed with the respective fixatives (1% PFA, 2% GA, 4F1G, A4B0.05P0, or A4B0.05P1) and subsequently cleared in CUBIC-1 buffer for 1 day. The extent of protein loss was quantified by measuring total protein content in the clearing solution using a NanoDrop spectrophotometer, with the CUBIC-1 buffer as blank. Protein loss was expressed as a percentage of the total protein normalized to the initial weight of the tissue slices prior to clearing.

### Transmittance measurement.

Transmittance were measured as previously described^23^. In brief, CUBIC-cleared myocardial sections (0.6 mm thick) were excised from the central region of the heart to minimize tissue heterogeneity among samples. Each slice was trimmed to fit within the wells of a 96-well plate and submerged in the minimum volume of clearing solution required for complete coverage. Optical absorbance was measured across a wavelength range of 450–750 nm using a Tecan Infinite^®^ M1000 plate reader, with multiarea scanning (5×5 grid per well). The central nine measurement areas (3×3 grid) were used for quantification to avoid edge artifacts caused by viscosity gradients in the RI medium. Absorbance readings were blank-corrected and converted to transmittance using the relationship *T*=10*^−A^*, where *AA*A is the measured absorbance. Each condition was tested with six biological replicates (n = 6).

### Cryoinjury.

Cryoinjury was performed according to established zebrafish protocols^50^. Briefly, 6–8-month-old adult fish were anesthetized in 0.04% tricaine (Sigma-Aldrich, St. Louis, MO) and positioned ventral side up in a moist sponge. A small incision was made in the thoracic wall using fine microdissection scissors to expose the ventricle. A stainless-steel probe pre-chilled in liquid nitrogen was gently applied to the ventricular surface until the metal warmed to room temperature. After injury, fish were transferred to fresh system water to recover, and gentle pipetting of water across the gills was used to facilitate reanimation.

### Myocardial infarction.

Myocardial infarction was induced by ligation of the left anterior descending (LAD) coronary artery in 3–4-week-old *Myh6-Cre/* + *;R26-tdTomato/* + mice. Animals were anesthetized via intraperitoneal injection of tribromoethanol (Avertin; 0.25 mg/kg; Sigma-Aldrich), intubated with a 22G endotracheal tube, and ventilated using a small-animal respirator. A thoracotomy was performed between the fourth and fifth ribs to expose the heart, and the LAD artery was ligated approximately 2 mm below the left atrial margin with a 7-0 nylon suture. The chest wall was then closed using a 6-0 nylon suture, and mice were maintained under a heat lamp until full recovery.

### Immunostaining of thick sections.

Decolorized tissue samples were permeabilized in 5% DMSO with 1% Triton X-100 in PBS for 2 days at room temperature (RT). Blocking was carried out for 6 h to 1 day at RT. Samples were then incubated with primary antibodies for 5–7 days, beginning at 1.2 μg ml^−1^ and gradually increasing to 3.5–5 μg ml^−1^. After extensive washing (six 1-h washes at RT, one overnight wash at 4 °C, and six additional 1-h washes at RT), samples were incubated with secondary antibodies for 3–7 days at dilutions ranging from 1:100 to 1:75 or 1:50. Following secondary staining, tissues were washed again for 1–3 days using the same regimen. The total staining volume was maintained at ≥1 ml per sample to ensure adequate antibody penetration throughout the tissue. Wheat germ agglutinin (WGA) and phalloidin staining were performed at a 1:300 dilution.

### Confocal Imaging.

Cleared samples were incubated in CUBIC-2 refractive index–matching solution overnight at room temperature (RT) to minimize optical heterogeneity. Samples were then transferred to 35-mm glass-bottom dishes (Grenier BioOne CELLview 627860)and imaged using a Zeiss LSM 880 confocal microscope equipped with a 10x/0.3 or 20x/0.8 objective. Three-dimensional image stacks were collected at Nyquist sampling, with pinholes set to 1.5 Airy units. Image tiles were collected with 10% overlap and stitched together using Zeiss Zen Black software.

### Light-sheet imaging.

Cleared samples were incubated overnight at room temperature in a 1:1 (w/v) mixture of low-melting agarose and CUBIC-2, then embedded in the same mixture. The embedded samples were further incubated overnight in CUBIC-2 at room temperature to equilibrate the refractive index. After solidification, samples were mounted on the sample holder using cyanoacrylate gel and imaged with a Zeiss Lightsheet 7 microscope equipped with a 5×/0.16 NA objective in a standard sample chamber (26 × 28 × 48 mm^3^). Images were acquired under bilateral illumination with 7 μm Z-step intervals. Image stacks were stitched in Zeiss ZEN or Arivis software for subsequent 3D rendering and quantitative analysis.

### Light-field imaging.

Cleared samples were immersed in CUBIC-1 and mounted in 35-mm glass-bottom dishes (Grenier BioOne CELLview 627860) under coverslips. Imaging was performed using a Zeiss LSM 910 Light Field 4D microscope under either a 10×/0.3 NA air objective (field size XYZ: 1444*1444*1712 μm^3^; voxel Size XYZ 2.8*2.8*18 μm^3^), 25×/0.8 NA water-immersion objective (field size XYZ: 585*585*278 μm^3^; voxel size XYZ 1.12*1.12*2.7 μm^3^), or a 40x/1.1 NA water-immersion objective (field size: 361*361*109 μm^3^; voxel size XYZ 0.7*0.7*0.9 μm^3^). Light-field volumes were reconstructed in Zeiss ZEN blue’s built-in Light Field 4D module using the default wave-optics model.

## Extended Data

**Extended Data Fig. 5 |:**
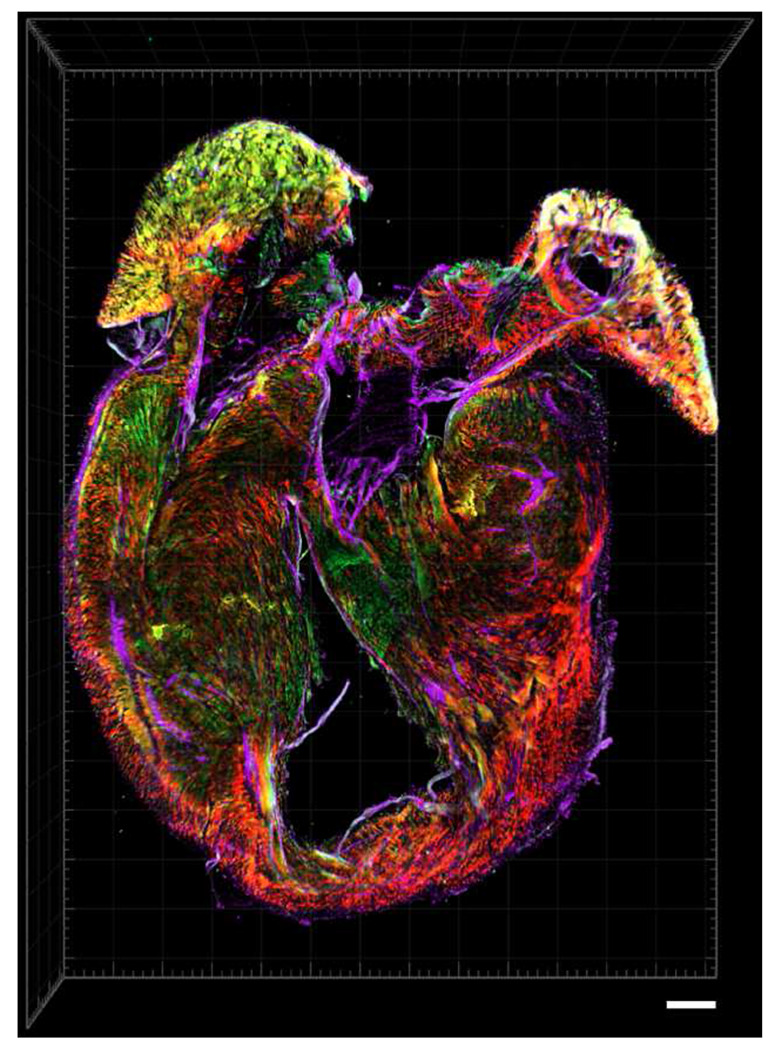
High-resolution volumetric rendering of a neonatal mouse heart. Representative LFM reconstruction of a cleared neonatal *Myh6-Cre/ +; R26-tdTomato/ +* heart stained with wheat germ agglutinin (WGA; magenta) to delineate extracellular matrix and phalloidin (green) to label filamentous actin. tdTomato expression (red) marks cardiomyocytes and preserves myocardial fiber orientation throughout the ventricular wall. The dataset illustrates the high contrast and structural continuity achievable after clearing submillimeter cardiac tissue, enabling visualization of cardiomyocyte alignment, trabecular networks, and atrioventricular junctional architecture. Scale bar, 500 *μ*m.

## Supplementary Material

This is a list of supplementary files associated with this preprint. Click to download.

• Supplementarysummarytable.xlsx

• Supplementaryvideos.zip
